# Supplementary material for: The Impact of Age and Vaccine Conspiracy Beliefs on COVID-19 Vaccine Uptake among United States Adults
Source: Vaccines (Basel). 2024 Jul 30;12(8):853. doi: 10.3390/vaccines12080853 (PMC11359852; doi:10.3390/vaccines12080853)
Supplement: Supplementary file 1 [file vaccines-12-00853-s001.zip › vaccines-3071620-supplementary.pdf]

Supplementary Material

Items from the original English version of the Vaccine Conspiracy Beliefs Scale (Shapiro et al., 2016; https://doi.org/10.1016/j.pvr.2016.09.001) that were used in an investigation by Furlan, Chin, Menounos, & Anselmi (2024).

1. Vaccine safety data is often fabricated.
2. Immunizing children is harmful and this fact is covered up.
3. Pharmaceutical companies cover up the dangers of vaccines.
4. People are deceived about vaccine efficacy.
5. Vaccine efficacy data is often fabricated.
6. People are deceived about vaccine safety.
7. The government is trying to cover up the link between vaccines and autism.

Participants are instructed to rate how much they agree with each statement on a scale that ranges from strongly disagree (1) to strongly agree (7).
